# Etiology of sexually transmitted infections among people living with HIV (PLWHIV) within meme division in Cameroon: seroprevalence and factors associated with transmission

**DOI:** 10.3389/fpubh.2025.1683257

**Published:** 2025-12-11

**Authors:** Bekindaka Ngemani Obase, Elvis Asangbeng Tanue, Awanakam Honore Awanakam, Achu Charlton Odape, Aboudou Habirou Kifouly, Forgu Esemu Livo, Biyeh Edwin Abi, Jude Daiga Bigoga, Nsagha Dickson Shey, Rose Leke Fomban, Mispa Yivala Mbanyamsig, Mitchell Morey, David Seidenfeld

**Affiliations:** 1 Union for African Population Studies, Accra, Ghana; 2 Pan African University Life and Earth Sciences Institute (Including Health and Agriculture), University of Ibadan, Ibadan, Nigeria; 3 Health Watch Cameroon, Yaoundé, Cameroon; 4 Immunology and Molecular Biology Laboratory of the Biotechnology Research Center, University of Yaoundé I, Yaoundé, Cameroon; 5 American Institute for Research, Arlington, VA, United States; 6 Department of Public Health and Hygiene, Faculty of Health Sciences, University of Buea, Buea, Cameroon; 7 Center for Research on Emerging and Re-emerging Diseases, Institute for Medical Research and Medicinal Plant Studies, Yaoundé, Cameroon; 8 Department of Biochemistry, Faculty of Science, University of Yaoundé I, Yaoundé, Cameroon; 9 Ejed Paramedical and Vocational Training School, Kumba, Cameroon; 10 Department of Animal Health and Production, University of Abomey-Calavi, Calavi, Benin; 11 Department of Biomedical Sciences, Faculty of Health Sciences, University of Buea, Buea, Cameroon; 12 Collage of Public Health, Texila American University-Guyana, Guyana, US Minor Outlying Islands

**Keywords:** prevalence, chlamydia, syphilis, hepatitis, HIV, Kumba, meme-division

## Abstract

**Introduction:**

Sexually transmitted infections are of great public health importance. The screening of these infections not being part of the routine healthcare package for PLWHIV means most of the infected individuals who are asymptomatic end up being undetected. This study aims to determine the seroprevalence of syphilis, chlamydia, and HBV among PLWHIV in Meme division. Additionally, it aimed to assess the factors associated with STI transmission.

**Methods:**

A hospital-based cross-sectional design was adopted that recruited a total of 364 PLWHIV from the urban and rural communities in Meme division from December 2024 to April 2025. About 4 mL of blood was collected and placed in a sodium citrated tube. The sample was used for ABO blood grouping, syphilis, hepatitis B, and chlamydia screening using serological assays. The data was analysed using SPSS version 25. Fisher’s exact test was used to determine the difference in proportions and logistic regression model was used to determine risk factors associated with STI transmission. *p*-values <0.05 were considered as statistically significant.

**Results:**

The overall seroprevalence of syphilis, HBV and chlamydia infection among PLWHIV from rural areas was 30/150 (20%) [95% CI 0.139–0.273], 13/150 (8.7%) [95% CI 0.047–0.143] and 6/150 (4.0%) [95% CI 0.014–0.085] while in urban area the seroprevalence was 41/214 (19.2%) [95% CI 0.141–0.250], 33/214 (15.4%) [95% CI 0.108–0.209] and 5/214 (2.3%) [95% CI 0.007–0.053] respectively. A couple of the individuals were infected with at least more than one STI. Multivariate analysis shows that females had a lower odd to STI infection (AOR = 0.468, 95% CI 0.252–0.867, *p* < 0.016), having multiple sexual partners had a lower odd to STI (AOR = 0.346, 95% CI 0.160–0.748, *p* < 0.007), bathing before and after sexual intercourse recorded a lower odd to STI (AOR = 0.458, 95% CI 0.272–0.772, *p* < 0.003) as well as those who reported having sex 1–3 times a week also had a lesser odd to STI (AOR = 0.526, 95% CI 0.296–0.935, *p* < 0.029).

**Conclusion:**

Sexually transmitted infection was high among PLWHIV in Meme division with individuals from rural communities having the highest prevalence of infections. The most dominant of these infections was syphilis followed by HBV and lastly Chlamydia.

## Introduction

Sexually transmitted infections, which comprise a variety of clinical syndromes that can be contracted and spread through sexual activity, are of great public health importance. Viral, bacterial, or parasitic organisms that cause STIs can spread from person to person by vaginal fluids, semen, or blood. It is estimated that over 1 million STIs are contracted every day (1). The screening of these infections not being part of the routine healthcare package for PLWHIV in Cameroon means most of the infected individuals who are asymptomatic ends up being undetected and this may lead to serious health complications including ART-drug resistance. HIV/AIDS pandemic continues to be a serious global public health issue. 84.2 million (64.0–113.0 million) persons have contracted HIV since the pandemic began, and roughly 40.1 million (33.6–48.6 million) have lost their lives to the virus (2, 3). Even though access to antiretroviral therapy (ART) has dramatically increased in Sub-Saharan Africa over the past ten years, the region is still the most severely affected by the HIV pandemic (3). In the West and Central African Subregion, Cameroon is among the nations with the highest HIV prevalence. The nation has a 2.9% HIV prevalence rate. Although there were 15,000 new cases of HIV in Cameroon in 2021, only 78% of PLWHIV are receiving antiretroviral therapy (3).

The illness is known to target the host immune system, impairing the body’s defences against opportunistic diseases like chlamydia, hepatitis, syphilis and gonorrhoea as well as a host of other infections. Sexually transmitted infections (STIs) are a major burden for people living with HIV (PLWHIV) in comparison to people without HIV. Behavioural patterns, biological vulnerabilities, and possible interactions between HIV and STIs are some of the factors associated with higher prevalence (4). These infections even though they are well established for their contribution in HIV transmission by penetrating protective mucosal barriers and attracting vulnerable immune cells (such as macrophages and CD4 T-helper cells) to the infection site, STIs aid in the spread of HIV (5). They provide an entry point into vulnerable cells.

Sexually transmitted diseases (STIs) tend to accumulate in core areas, also known as risk spaces, which might be spatially delimited (e.g., drug-using areas, neighbourhood level, local sexual partner selection). Given that these core locations are typically located in low-socioeconomic-status (SES) urban neighbourhoods, it is plausible that sociocultural factors of health influence the STI concentrated spatial pattern (6). For example, urban environments have been linked to gonorrhoea by several number of socio-cultural risk factors, including those at the individual level (e.g., SES) and the community level (e.g., country or state) (prevalence of infection (7), percentage urbanicity (8), percentage neighbourhood instability (9), gender imbalance with more women than men, low social capital (10, 11), and high percentage Black or Hispanic populations) (12, 13). In a sexual network, for example, low SES can hinder prompt access to STI services, lengthening the incubation period and eventually raising the transmission rate.

Observations from previous studies conducted in Bangladesh reported that residence location (urban–rural) plays a significant role in STI transmission with individuals in rural areas having a much higher prevalence (11.6%) compared to those from urban areas (14). In Cameroon, even though few studies have been conducted looking at the prevalence of these infections (1, 15, 16), studies assessing the etiological causes of STIs among PLWHIV are uncommon, particularly in remote areas like Kumba where public awareness of these infections is low. Therefore, it is essential to comprehend the prevalence of STIs in PLWHIV to effectively address and manage these infections and the health risks they pose. The purpose of this research is to ascertain the prevalence of HBV, syphilis, and chlamydia in PLWHIV from both urban and rural locations. It also sought to evaluate the risk factors for STI transmission among the study populations.

## Materials and methods

### Study design

A hospital based cross-sectional study was adopted, recruiting participants coming from the urban and rural areas seeking for health care services at the three health facilities (Presbyterian hospital Buea Road Kumba, District Hospital Kumba town/PMI Kumba and CMA Ntam Fiango) with HIV treatment centres offering HIV treatment services to patients from all the different localities in meme division. Enrolment of participants started in December 2024 and ended in April 2025.

### Study area

The study was conducted in the southwest region of Cameroon in the Kumba health district, which is a part of the Meme Division with a population of about 326,734 and a surface area of roughly 3,105 km^2^, the division has Kumba as its headquarters. Five sub-divisions comprise the division: Kumba I, II, III, Mbonge, and Konye. Numerous medical facilities, including government health Centre’s and private clinics, are in the area, including the Regional Hospital Annex-Kumba, District Hospital Kumba town, CMA Ntam, Presbyterian Hospital Buea Road Kumba, Catholic Hospital Fiango-Kumba, Kossala Integrated Health Center-Kumba, and many more.

### Ethical consideration

Ethical clearance for this study was obtained from the institutional review board of the Southwest regional ethics committee at the regional delegation for public health (Ref N^o^: 017/CRERSH/SW/C/08/2024). Administrative approval was gotten from the regional delegation for public health Southwest region (Ref N^o^: P42/MINSANTE/SWR/RDPH/RCB. PT/66/714) as well as the directors/heads of all the health institutions concerned in the study. Data collected during this study are strictly confidential, accessible only to the principal investigator and his assistant. Future studies aiming to use this data will sign an memorandum of understanding (MoU) testifying to maintain participant secrecy.

### Sample size determination

A total of 364 participants were recruited for the study. However, the calculated sample size was 174 including 10% attrition rate using the Cochrane’s formula (17). This was calculated using a prevalence of 11.6% syphilis among PLWHIV in a study conducted in Yaoundé (18). Z value at 95% confidence interval was calculated to be 1.96, q was 1-p and margin of error (d) was 0.05.


$$n=Z^{2}\mathrm{Pq}/d^{2}$$

$$n=1.96^{2}\;x\;0.116\;(1–0.116)/0.052$$

n=3.8416x0.116(0.884)/0.0025=158+10%attrition rate=174

### Recruitment of study participants

The survey recruited PLWHIV seeking HIV treatment at the HIV treatment centres of the three different health facilities concerned with the enrolment of participants. Participants recruited were from all the different localities within meme division in the southwest region such as Matoh, Mbonge, Mamfe road, Kumba, Mbalangi, Banga Bakundu, Kombone mission, Big Ekombe, Bai panya, Bombele, Kake I and II, Bai Kuke, Mabonji, Ikiliwindi, Bole Bakundu, Kwa-kwa, small Ekombe, Weme, Konye, Ngongo etc. All these areas and many more fall within the Meme division which is our research area of interest.

### Eligibility criteria

#### Inclusion criteria

All participants living with HIV infection who are age 18 years and above were recruited. It enrolled both males and females living within meme division and took into consideration only the participants seeking HIV treatment at the HIV treatment centres of the Health Institutions concerned.

#### Exclusion criteria

The study did not take into consideration participants who were below 18 years of age, those who were seeking HIV treatment at other health facilities/treatment centres other than the sites for our study, and it did not consider participants seeking for treatment at the different study sites but are not permanently living within meme division.

### Sampling technique

A probability proportionate to size sampling method was used to recruit participants into the study at the three different health facilities considered for the enrolment of participants (Table 1). The number of PLWHIV from urban and rural communities within the Meme division coming for treatment in each of the health facilities was gotten from the different hospital statistics. This was used to determine the probability of participants to be enrolled for each site which when multiplied by the calculated sample size, gave us the number of individuals to recruited in each of the different health facilities or probability sample size (pss).

**Table 1:** Number of participants to be recruited from the three different health institutions within the Kumba Health district area.

	Presbyterian Hospital Kumba			District hospital Kumba town			CMA Ntam Fiango Kumba		
	Population PLWHIV	Probability	PSS	Population PLWHIV	Probability	PSS	Population PLWHIV	Probability	PSS
Urban	966	0.460	80	598	0.620	108	545	0.542	94
Rural	1,134	0.540	94	373	0.384	66	459	0.457	80
Total	2,100	1	174	971	1	174	1,004	1	174

### Sociodemographic, economic, behavioural and clinical baseline data of the study population

A well-structured and tested questionnaire was used to collect the appropriate data needed to capture the different variables of interest from our study participants such as: Age, Sex, marital status, level of education, occupation, estimated income per month, toilet type used, hygienic condition of the toilets, number of person living in a household, health facility location from place of resident, pregnancy, tribe, sexual behaviour such as; having multiple sex partners, sex frequency per week, use of condom during sexual intercourse, sex preference, frequency of ART uptake, drinking of alcohol, smoking, hygienic condition before and after having sex. Also, the clinical parameters taken into consideration were High blood pressure, diabetes, discharges, itches, sore/bumps, abdominal pain, rhesus type, and ABO blood group.

#### Sample collection, transportation, processing and storage

A total of about 4 mL of venous blood was collected from each participant in a sterile sodium citrate tube using a 5 mL syringe for the different serological assays to be performed during laboratory analysis of the sample. The collected samples were transported to the laboratory of the district hospital Kumba town using a sample transportation box. Upon arrival at the laboratory, quality control check was done to check for haemolysis and missing data. The samples were processed and stored at a + 4 °C refrigerator in preparation for the different downstream serological analyses. The whole blood sample was centrifuged at 2000 rpm/5 min after performing the full blood count and blood grouping analysis, to separate the plasma from the red cells. The plasma was aliquoted into cryotubes and stored at −20 °C

## Laboratory procedures

Using the stored and processed samples, the following analyses were performed:

### Blood grouping

The whole blood sample stored at +4 °C was used for the ABO and rhesus blood grouping. Before the start of the assay, reagents and samples were brought to room temperature (18–25 °C). A wax pen was used to divide the slide into appropriate numbers of divisions. Using the provided dropper in the kit box, one drop (40 ± 10 μL) of each reagent was placed onto its corresponding division on the slide. 25 μl of the precipitated cells was added next to each drop of reagents. The reagent and the cells were mixed using a clean stirring stick over an area with a diameter of approximately 20–40 mm. The slide was incubated at room temperature (18–25 °C) without stirring for 30 s. The slide was held and gently rocked for 3 min and observed macroscopically for any agglutination. Reaction was read immediately, and the presence of agglutination was reported as positive. The product used was blood grouping antisera from Rapid Lab®.

### Syphilis, chlamydia and hepatitis B (HBsAg-2) testing

The plasma from the whole blood samples stored at −20 °C were used to perform serological assay for syphilis [DiaSpot: *Lot #:* −*231229*], chlamydia IgG/IgM [DiaSpot: *Lot #:* −*240408*] and HBV [Determine^Th^ (Abbott: *Lot #: 0000755655*)] using the dip-stick method (19) following the manufacturer instruction. The results were interpreted qualitatively as non-reactive (negative) or reactive (positive).

### Data quality assurance

#### Data quality management

All laboratory materials such as rapid test kits for serological testing, blood grouping reagent, test tubes, sample transporting system were checked by an experienced laboratory professional to ensure that they are not expired as well as performing well. Kits and reagents were stored in the appropriate storage conditions as recommended by the manufacturers. Samples collected were checked for haemolysis and labelling. Those hemolyzed were sorted and discarded immediately. Sample collection was done by well-trained laboratory professionals. Questionnaires with missing information were sorted out and participants were called on phone to address the missing issues. Those who did not comply were excluded from the study.

### Data processing and analysis

The data collected from the study participants were entered into Kobo tool collect software. The MS Excel spread sheet developed was then screened for any irregularities. The data was then analysed using SPSS (Statistical package for the social sciences) version 25.0 and Datawaraper was used to develop descriptive charts. Descriptive analysis was used to show the seroprevalence of STIs while Fisher’s exact test was used to show difference in the proportion of STI seroprevalence between participants who came from the urban and rural communities. Multivariate analysis was performed using logistic regression to determine factors associated with STI transmission among our study population. *p*-values that were less than 0.05 were statistically significant.

### Definition of terminologies

PLWHIV: people living with HIV; STI infected: individuals infected with one or more of the STIs of interest; Mono-infected: individuals infected with just one of the three STIs (syphilis, chlamydia, HBV); Dual-infected: individual infected with two of the STIs; Poly-infected: individuals infected with all three of the STIs; prevalence: proportion of a population infected/ showing a particular characteristic within a given period; PSS: probability sample size.

## Results

### Socio-demographic characteristics of the study population

Table 2 shows the age range of the study population with the mean age for the PLWHIV from the urban areas being 43.90 years (range: 18–82 years) and that from the rural communities being 46.31 years (range: 20–77 years). Majority of the participants in both groups were between the age of 25 − 45 years. Most of the participants were females from both the rural 120/150 (80%) and urban 174/214 (81.3%) communities. There was a significant difference (*p* < 0.0001) in the level of education in our study population from the urban and rural area. Most of the individuals from rural communities had just primary education 95/150 (63.3%) while for the urban community, most are having secondary education 118/214 (55.1%) with a hand full of the participants in both groups being married. There was a significant difference (*p* < 0.0001) also in both groups in terms of occupation. Most of the participants in the rural communities were farmers 109/150 (72.7%) while in the urban area, most of them do business 104/214 (48.6%). The types of toilets used also at the urban and rural communities had a significance difference (*p* < 0.0001) with most of the individuals in the urban area using flushing toilets 112/214 (52.3%) while most of the participants 135/150 (90.0%) from the rural communities were using the pit toilets with a significant (*p* < 0.0001) disproportionate share of the participants in the urban and rural communities testifying of washing their toilets less than 3-times in a week. There was a significant difference (*p* < 0.0001) also in the hospital facility location from residents. In the rural communities, most of the individuals 80/150 (53.3%) stay 1–2 h drive away from a health facility, while in the urban area most of them 199/214 (93.0%) stay just less than 1 h drive from a health facility.

**Table 2:** Sociodemographic characteristics of the study population.

Variables	Category	PLWHIV-Rural		PLWHIV- Urban		*p*-value
		Frequency	(%)	Frequency	(%)	
Age group (years)	18–24	03	2.0	09	4.20	0.330
	25–45	77	51.3	118	55.1	
	≥ 46	70	46.7	87	40.7	
Sex	Male	30	20.0	40	18.7	0.788
	Female	120	80.0	174	81.3	
Marital status	Single	54	36.0	101	47.2	0.068
	Married	85	56.7	95	44.4	
	Divorced	02	1.3	01	0.50	
	Widowed	09	6.0	17	7.90	
Level of education	Not educated	16	10.7	08	3.70	0.0001
	Primary	95	63.3	79	36.9	
	Secondary	36	24.0	118	55.1	
	Tertiary	03	2.0	09	4.20	
Occupation	Unemployed	10	6.7	26	12.1	0.0001
	Farmer	109	72.7	47	22.0	
	Private employee	05	3.3	12	5.60	
	Business	20	13.3	104	48.6	
	Civil servant	05	3.3	08	3.70	
	Students	01	0.7	17	7.90	
Estimated income (FCFA)	< 30,000	120	80.0	155	72.4	0.392
	31,000–50,000	07	4.70	12	5.60	
	51,000–100,000	20	13.3	38	17.8	
	>100,000	03	2.00	09	4.20	
Toilet types used	Flushing toilet	15	10.0	102	47.7	0.0001
	Pit toilet	135	90.0	112	52.3	
Number of times the toilet is cleaned per week	<3	129	86.0	146	68.2	0.0001
	> 2	21	14.0	68	31.8	
Number of persons in Household	1–5	97	64.7	150	70.1	0.305
	>5	53	35.3	64	29.9	
Religion	None	03	2.0	04	1.90	0.451
	Christian	147	98.0	207	96.7	
	Muslim	00	0.00	03	1.40	
Health facility location from residence	< 1 h	53	35.3	199	93.0	0.0001
	1–2 h	80	53.3	13	6.10	
	> 2 h	17	11.3	02	0.90	
Pregnant	Yes	05	3.30	04	1.90	0.497
	No	145	96.7	210	98.1	

### Behavioural characteristics of the study population

There was a significant difference (*p* < 0.025) in the population of individuals who bath before and immediately after having sex from the rural and urban communities. Most of the individuals 91/150 (60.7%) in the rural communities testified of bathing while in the urban community, most of them 110/214 (51.4%) do not bath before or even after having sexual intercourse (Table 3).

**Table 3:** Behavioural characteristics of study participants.

Variables	Category	PLWHIV-Rural		PLWHIV-Urban		*p*-value
		Frequency	(%)	Frequency	(%)	
Having other sexual partners	Yes	12	8.00	27	12.60	0.173
	No	138	92.0	187	87.40	
Use of condom	Yes	14	9.4	26	12.1	0.496
	No	136	90.7	188	87.9	
Frequency of sexual intercourse/week	0	28	18.7	58	27.1	0.142
	1–3 times	117	78.0	147	68.7	
	>3 times	05	3.30	09	4.20	
Sexual preference	Bisexual	0	0.0	03	1.40	0.296
	Homosexual	02	1.3	01	0.50	
	Heterosexual	148	98.7	210	98.1	
Frequency of ART uptake	Frequent	121	80.7	182	85.0	0.318
	Skip	29	19.3	32	15.0	
Drinking of alcohol	Yes	111	74.0	142	66.4	0.133
	No	39	26.0	72	33.6	
Smoking/snuffing	Yes	05	3.30	04	1.90	0.497
	No	145	96.7	210	98.1	
Bath before and immediately after sexual intercourse	Yes	91	60.7	104	48.6	0.025
	No	59	39.3	110	51.4	

### Clinical characteristics of the study participants

There was no significant difference in the clinical characteristics of the study population from both groups. However, it was observed that there was a slim significant difference (*p* > 0.072) in the number of individuals with high blood for both groups. Just 3/150 (2.0%) of the participants in the rural areas had high blood pressure while for the urban area, 13/214 (6.10%) suffered from high blood pressure with most of the individual from the rhesus positive blood group and the “O” blood group type (Table 4).

**Table 4:** Clinical characteristics of study participants.

Variables	Category	PLWHIV-Rural		PLWHIV-Urban		*p*-value
		Frequency	(%)	Frequency	(%)	
High blood	Yes	03	2.0	13	6.10	0.072
	No	147	98.0	201	93.9	
Diabetes	Yes	0	0.0	02	0.90	0.514
	No	150	100	212	99.1	
Discharges	Yes	06	4.0	16	7.50	0.188
	No	144	96.0	198	92.5	
Itches	Yes	03	2.00	10	4.70	0.253
	No	147	98.0	204	95.3	
Sore/bumps	Yes	01	0.70	04	1.90	0.653
	No	149	99.3	210	98.1	
Abdominal pain	Yes	01	0.70	03	1.40	0.646
	No	149	99.3	211	98.6	
Rhesus type	ABO Rh-	15	10.0	19	8.90	0.718
	ABO Rh+	135	90.0	195	91.1	
ABO Blood group	A	41	27.3	69	32.2	
	AB	04	2.70	02	0.90	0.480
	B	26	17.3	37	17.3	
	O	79	52.7	106	49.5	
HBV vaccine	Yes	02	1.30	03	1.40	1.000
	No	148	98.7	211	98.6	

### Seroprevalence of chlamydia, syphilis and HBV infections among people living with HIV

**Figure 1:**
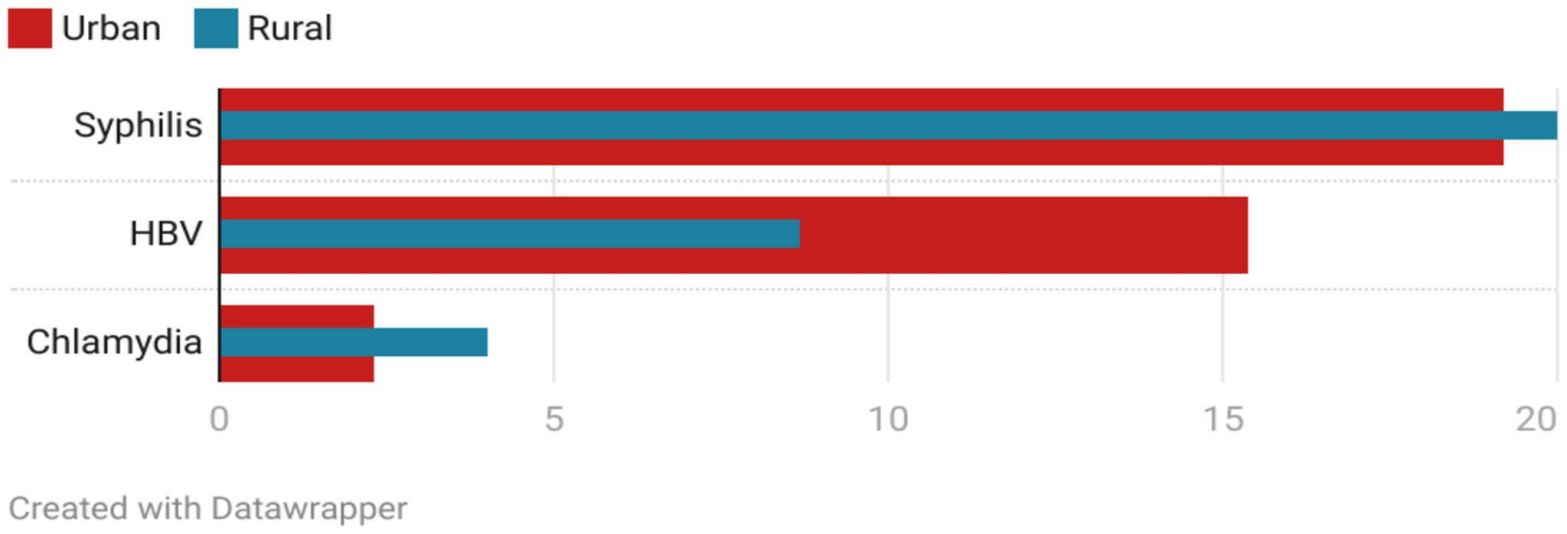
General prevalence of chlamydia, syphilis and Hepatitis-B infection among PLWHIV from urban and rural communities.

The general seroprevalence of STI was higher in the rural communities than in the urban communities except for HBV which was higher in urban areas than rural areas (Figure 1). In the rural communities, the seroprevalence of syphilis was 30/150 (20%) [95% CI 0.139–0.273] in the rural communities, while in the urban area the seroprevalence was 41/214 (19.2%) [95% CI 0.141–0.250]. This was followed by Hepatitis B virus with a seroprevalence of 13/150 (8.7%) [95% CI 0.047–0.143] and 33/214 (15.4%) [95% CI 0.108–0.209] respectively. Chlamydia was the lowest with 6/150 (4.0%) [95% CI 0.014–0.085] in the rural communities and 5/214 (2.3%) [95% CI 0.007–0.053] in the urban communities. IgG was 2/214 (0.93%) [95% CI 0.001–0.033], IgM 2/214 (0.93%) [95% CI 0.001–0.033] and IgM + IgG 1/214 (0.46%) [95% CI 0.0001–0.0258] among participants from urban areas. While for participants from rural areas IgM was 5/150 (3.33%) [95% CI 0.0109–0.0761] and IgG 1/150 (0.66%) [95% CI 0.0002–0.0366].

### Seroprevalence of STIs co-infection among people living with HIV infection

**Figure 2:**
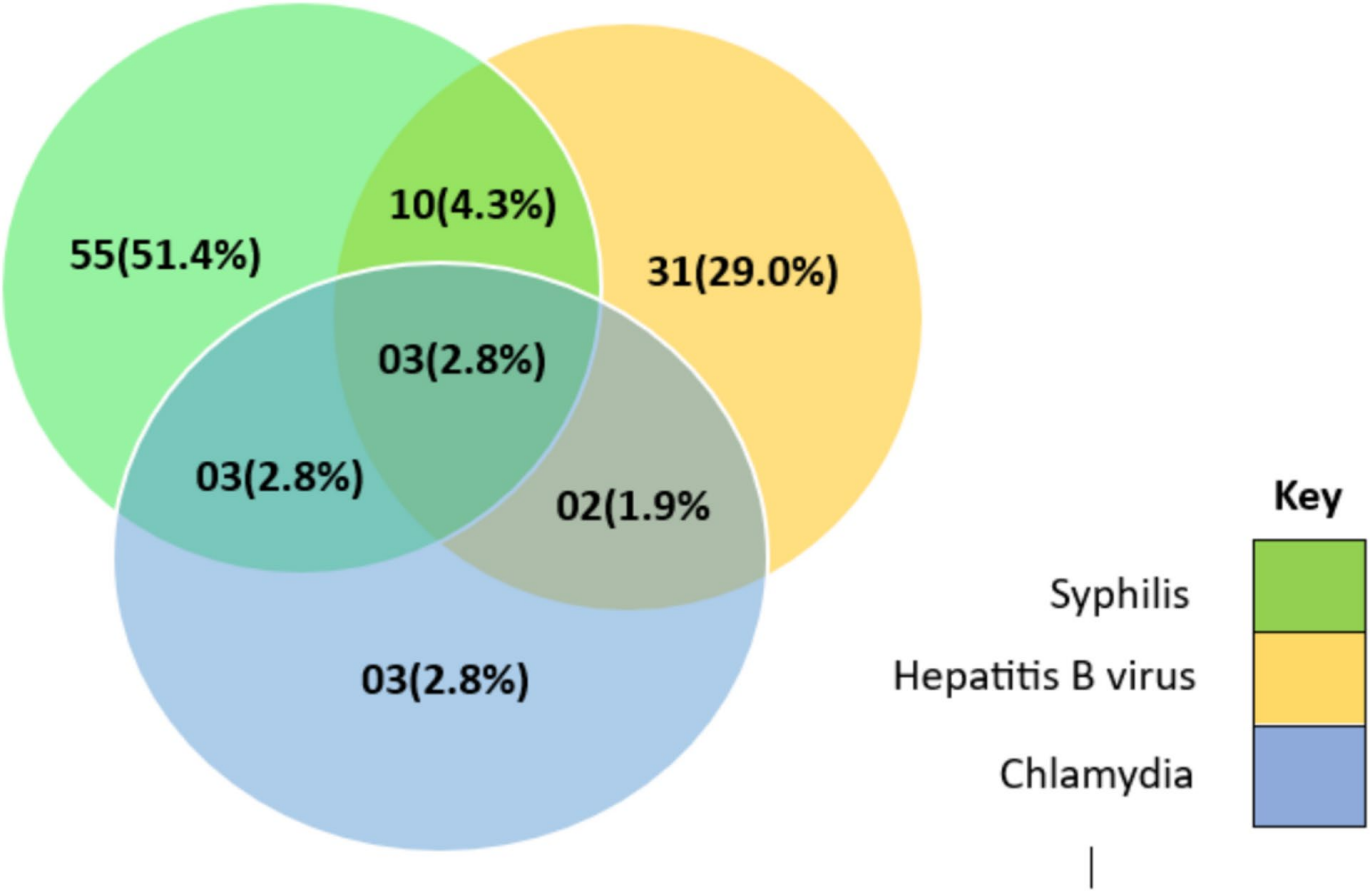
Percentage seroprevalence of co-infection of the sexually transmissible infections among PLWHIV coming from the rural and urban communities.

Mixed infection was common among the study population (Figure 2). 10/364 (4.3%) [95% CI 0.013–0.049] of the participants were infected with both HBV and syphilis, 3/364 (2.8%) [95% CI 0.001–0.023] was infected with chlamydia and syphilis, 2/364 (1.9%) [95% CI 0.001–0.019] was infected with chlamydia and HBV while 3/364 (2.8%) [95% CI 0.001–0.023] was infected with all three infections. Nevertheless, a greater percentage of the individuals from the urban area were mono-infected 56/89 (62.9%) [95% CI 0.520–0.729] compared to those from the rural areas, Bi-infection was more among those from the rural areas 8/15 (53.3%) [95% CI 0.265–0.787] compared to the urban area and all triple infections (poly-infection) 3/3 (100%) [95% CI 0.292–1.00] was recorded among those from the urban area (Table 5).

**Table 5:** Percentage proportion of single, double and triple sexually transmitted infections among PLWHIV infection from the rural and urban communities.

	Mono-infected	Bi-infected	Poly-infected
	Positive (%)	Positive (%)	Positive (%)
Rural area	33 (37.1)	08 (53.3)	0 (0.0)
Urban area	56 (62.9)	07 (46.7)	03 (100)

### The seroprevalence of STI among PLWHIV coming from the urban and rural communities in meme division

Individuals’ positive with at least one or more of the three STIs were considered as STI positive. There was significant association between educational level and STI prevalence (*p* < 0.001) with non-educated individuals who came from rural areas having the highest seroprevalence of STI 3/5 (60%) [95% CI: 0.146–0.947] when compared to those who came from urban areas. However, among the individuals who came from urban communities, those with tertiary education recorded the highest prevalence 1/1 (100%) [95% CI: 0.025–1.000] when compared to those from rural communities. The type of occupation also has a significant association (*p* < 0.0001) with STI prevalence. Farmers from rural communities recorded the highest seroprevalence of STI 30/47 (63.8%) [95% CI: 0.485–0.773] when compared to those from the urban communities. Nevertheless, civil servants and students recorded the highest seroprevalence of 02/2 (100%) [95% CI: 0.158–1.000] and 04/4 (100%) [95% CI: 0.397–1.000] among participants from the urban and rural communities. The toilet type used among individuals from both communities also had an association (*p* < 0.0001) in the distribution of STI among PLWHIV. Majority 61 (80.3%) of those in urban areas living < 1 h drive from a health facility tested positive for STI, while in the rural communities, 20 (80.0%) of those living 1–2 h drive away from the hospital in rural communities recorded the highest prevalence of STI. Also, those living more than 2 h away from a health facility in rural areas recorded the highest prevalence of 6 (100%) (Table 6).

**Table 6:** Proportion difference in the prevalence of sexually transmissible infections among PLWHIV from rural and urban community’s vs. sociodemographic factors.

Variables	Category	STI-Rural		STI-Urban		*p*-value
		Positive	(%)	Positive	(%)	
Age group	18–24 years	0	0.0	03	100	0.201
	25–45 years	22	44.9	27	55.1	
	≥ 46 years	19	34.5	36	65.5	
Sex	Male	10	33.3	20	66.7	0.659
	Female	31	40.3	46	59.7	
Marital status	Single	15	34.1	29	65.9.	0.090
	Married	25	43.9	32	56.1	
	Divorced	1	100	0	0.0	
	Widowed	0	0.0	05	100	
Level of education	Non-educated	03	60	02	40	*0.001
	Primary	28	53.8	24	46.2	
	Secondary	10	20.4	39	79.6	
	Tertiary	0	0.0	01	100	
Occupation	Unemployed	01	11.1	08	88.9	*0.0001
	Farmer	30	63.8	17	36.2	
	Business	09	21.4	33	78.6	
	Civil servant	0	0.0	02	100	
	Students	0	0.0	04	100	
	Private	05	3.3	12	5.6	
Estimated income (FCFA)/Month	< 30,000	32	39.0	50	61.0	0.968
	31,000–50,000	07	38.9	11	61.1	
	51,000–10,000	01	25.0	03	75.0	
	>100,000	01	33.3	02	66.7	
Toilet types used	Flushing.t	04	10.5	34	89.5	*0.0001
	Pit toilet	37	53.6	32	46.4	
Number of times the toilet is cleaned per week	<3	36	42.9	48	57.1	0.090
	> 2	05	21.7	18	78.3	
Persons per Household	1–5 years	23	33.3	46	66.7	0.212
	>5 years	18	47.4	20	52.6	
Religion	None	02	50.0	02	50.0	0.495
	Christian	39	39.0	61	61.0	
	Muslim	0	0.0	03	100	
Health facility location from residence	< 1 h drive	15	19.7	61	80.3	*0.0001
	1–2 h drive	20	80.0	05	20.0	
	>2 h drive	06	100	0	0.0	
Pregnancy	Yes	0	0.0	0	0.0	---
	No	41	38.3	66	61.7	

### Proportion difference in the seroprevalence of STI vs. behavioural characteristics

Table 7 shows the seroprevalence of STI associated with the behavioural characteristics of the participants from the rural and urban communities. It was observed that there was no statistical difference across all the parameters investigated among participants from rural and urban communities.

**Table 7:** Proportion difference in the prevalence of STI among PLWHIV from rural and urban community’s vs. behavioural characteristic of the study population.

Variables	Category	STI-Rural		STI-Urban		*p*-value
		Positive	(%)	Positive	(%)	
other sexual partners	Yes	06	33.3	12	66.7	0.792
	No	35	39.3	54	60.7	
Use of condom	Yes	06	42.9	08	57.1	0.772
	No	35	37.6	58	62.4	
Frequency of sexual intercourse/week	0	07	24.1	22	75.9	0.209
	1–3 times	32	43.8	41	56.2	
	>3 times	02	40.0	03	60.0	
Sexual preference	Bisexual	0	0.0	01	100	1.000
	Homosexual	40	38.5	64	61.5	
	Heterosexual	01	50.0	01	50.0	
Frequency of ART uptake	Frequent	31	34.4	59	65.6	0.100
	Skip	10	58.8	07	41.2	
Drinking of alcohol	Yes	33	44.0	42	56.0	0.083
	No	08	25.0	24	75.0	
Smoking/snuffing	Yes	01	33.3	02	66.7	1.000
	No	40	38.5	64	61.5	
Bath before and immediately after sexual intercourse	Yes	28	32.5	39	67.5	0.413
	No	13	41.8	27	58.2	

### Proportion difference in the seroprevalence of STI vs. clinical characteristics of the study population

There was no statistical difference in the seroprevalence of STI across the clinical parameters investigated in our study population as can be seen in Table 8.

**Table 8:** Proportion difference in the prevalence of STI among PLWHIV from rural and urban community’s vs. clinical characteristic of the study population.

Variables	Category	STI-Rural		STI-Urban		*p*-value
		Positive	(%)	Positive	(%)	
High blood	Yes	01	25.0	03	75.0	0.659
	No	40	38.8	63	61.2	
Diabetes	Yes	0	0.0	0	0.0	---
	No	41	38.3	66	61.7	
Discharges	Yes	02	28.6	05	71.4	0.705
	No	39	39.0	61	61.0	
Itches	Yes	0	0.0	02	100	0.523
	No	41	39.0	64	61.0	
Sore/bumps	Yes	0	0.0	01	100	1.000
	No	41	38.7	65	61.3	
Abdominal pain	Yes	0	0.0	0	0.0	---
	No	41	38.3	66	61.7	
Rhesus type	ABO Rh-	04	50.0	04	50.0	0.708
	ABO Rh+	37	37.4	62	62.6	
ABO Blood group	A	33	41.3	47	58.8	
	AB	04	80.0	01	20.0	0.291
	B	18	38.3	29	61.7	
	O	54	43.2	71	56.8	
HBV vaccine	Yes	01	25.0	03	75.0	0.383
	No	108	42.7	145	57.3	

### Factors associated with the transmission of sexually transmitted infection among PLWHIV in meme division

Multivariate analysis shows that females have a lesser chance of being infected with STI compared to men (AOR = 0.468, 95% CI 0.252–0.867, *p* < 0.016) (Table 9).

**Table 9:** Shows the factors associated with the transmission of sexually transmitted infection among PLWHIV.

Variables	Category	Negative (%)	Positive (%)	COR [95% CL-]	*p*-value	AOR [95% CL-]	*p*-value
Age group	18–24 years	9 (75.0)	3 (25.0)	1			
	25–45 years	146 (74.9)	49 (25.1)	0.618 [0.161–2.22]	0.618	0.656 [0.135–3.185]	0.607
	≥ 46 years	102 (65.0)	55 (35.0)	0.622 [0.393–0.987]	0.622	0.665 [0.394–1.122]	0.126
Sex	Male	40 (57.1)	30 (42.1)	1			
	Female	217 (73.8)	77 (26.2)	0.473 [0.276–0.812]	0.007	0.468 [0.252–0.867]	*0.016
Marital status	Single	111 (71.6)	44 (28.4)	1			
	Married	123 (68.3)	57 (31.7)	2.100 [0.157–28.02]	0.575	1.448 [0.082–25.612]	0.801
	Divorced	2 (66.7)	1 (33.3)	1.946 [0.699–5.423]	0.203	1.566 [0.504–4.870]	0.438
	Widowed	2 1(80.8)	5 (19.2)	1.665 [0.591–4.691]	0.335	1.750 [0.570–5.369]	0.328
Level of education	Non-educated	19 (79.2)	5 (20.8)	1			
	Primary	122 (70.1)	52 (29.9)	2.895 [0.299–28.070]	0.359	3.286 [0.260–41.606]	0.358
	Secondary	105 (68.2)	49 (31.8)	4.689 [0.590–37.256]	0.144	4.360 [0.423–44.959]	0.216
	Tertiary	11 (91.7)	1 (8.3)	5.133 [0.645–40.885]	0.122	4.436 [0.455–43.264]	0.200
Occupation	Unemployed	27 (75)	9 (25)	1			
	Farmer	109 (69.9)	47 (30.1)	1.294 [0.565–2.962]	0.317	1.564 [0.569–4.302]	0.386
	Business	82 (66.1)	42 (33.9)	1.537 [0.663–3.563]	0.481	1.639 [0.599–4.463]	0.337
	Civil servant	11 (84.6)	2 (15.4)	0.545 [0.101–2.941]	0.542	0.579 [0.063–5.337]	0.629
	Private	1 (33.3)	2 (66.7)	0.643 [0.150–2.761]	0.552	1.046 [0.182–5.995]	0.960
	Students	14 (77.8)	4 (22.2)	0.857 [0.224–3.284]	0.822	0.704 [0.146–3.383]	0.661
Estimated income (FCFA)/month	< 30,000	193 (70.2)	82 (29.8)	1			
	31,000–50,000	40 (69.0)	18 (31.0)	0.628 [0.202–1.948]	0.721	1.624 [0.234–11.266]	0.624
	51,000–10,0000	15 (78.9)	4 (21.1)	1.059 [0.574–1.956]	0.854	0.759 [0.377–1.530]	0.441
	>100,000	9 (75.0)	3 (25.0)	0.785 [0.207–2.972]	0.420	0.782 [0.201–3.048]	0.724
Toilet types used	Flushing toilet	79 (67.5)	38 (32.5)	1			
	Pit toilet	178 (72.1)	69 (27.9)	1.241 [0.771–1.998]	0.375	1.492 [0.806–2.763]	0.203
Number of times the toilet is cleaned per week	<3	191 (69.5)	84 (30.5)	1			
	>2	66 (74.2)	23 (25.8)	1.262 [0.736–2.165]	0.398	1.054 [0.562–1.977]	0.871
Persons per Household	1–5 years	178 (72.1)	69 (27.9)	1			
	>5 years	79 (67.5)	38 (32.5)	0.806 [0.500–1.298]	0.375	0.840 [0.488–1.444]	0.527
Religion	None	3 (42.9)	4 (57.1)	1			
	Christian	254 (71.8)	100 (28.2)	0.295 [0.065–1.343]	0.295	0.268 [0.045–1.606]	0.150
	Muslim	0 (0.0)	3 (100.0)	1,211,606,132 [0.00--]	0.999	740225559.0 [0.000]	0.999
Health facility location from residence	< 1 h drive	176 (69.8)	76 (30.2)	1			
	1 – 2 h drive	68 (73.1)	25 (26.9)	0.936 [0.343–2.554]	0.897	0.771 [0.242–2.455]	0.660
	>2 h drive	13 (68.4)	6 (31.6)	0.797 [0.273–2.323]	0.677	0.636 [0.203–1.994]	0.438
Pregnancy	No	248 (69.9)	107 (30.1)	1			
	Yes	9 (100.0)	0 (0.0)	69699980.1 [0.000-]	0.999	516091508.5 [0.000]	0.999
Location of resident	Rural	109 (29.9)	41 (11.3)	1			
	Urban	148 (40.7)	66 (18.1)	0.843 [0.532–1.338]	0.470	0.961 [0.481–1.917]	0.909

### Behavioural factors which influence the transmission of STI among PLWHIV in meme division

Multivariate analysis shows that three out of the eight parameters showed some level of association with STI transmission. The analysis revealed that participants having multiple sexual partners have lower chances of contracting STI (AOR = 0.346, 95% CI 0.160–0.748, *p* < 0.007). After adjusting for frequency of sexual intercourse per week, the odd of STI among individuals reported to having sex 1–3 times a week appears to be low (AOR = 0.526, 95% CI 0.296–0.935, *p* < 0.029). While having a bath before and after sexual intercourse lowers the chances to having STI (AOR = 0.458, 95% CI 0.272–0.772, *p* < 0.003) (Table 10).

**Table 10:** Behavioural characteristics associated with the transmission of sexually transmitted infections among PLWHIV.

	Category	Negative (%)	Positive (%)	COR [95% CL]	*p*-value	AOR [95% CL]	*p*-value
Have multiple sexual partners	No	236 (72.6)	89 (27.4)	1		1	
	Yes	21 (53.8)	18 (46.2)	0.440 [0.224–0.864]	0.017	0.346 [0.160–0.748]	*0.007
Use of condom	No	231 (71.3)	93 (28.7)	1		1	
	Yes	26 (65.0)	14 (35.0)	0.748 [0.374–1.495]	0.411	0.908 [0.424–1.944]	0.804
Frequency of sexual intercourse/week	0	57 (66.3)	29 (33.7)	1		1	
	1–3 times	191 (72.3)	73 (27.7)	0.916 [0.281–2.984]	0.884	0.526 [0.296–0.935]	*0.029
	>3 times	09 (64.3)	05 (35.7)	0.688 [0.223–2.121]	0.515	---	
Sexual preference	Bisexual	02 (66.7)	01 (33.3)	1		1	
	Heterosexual	254 (70.9)	104 (29.1)	0.250 [0.008–7.452]	0.423	0.260 [0.007–9.223]	0.459
	Homosexual	01 (33.3)	02 (66.7)	0.018 [0.018–2.282]	0.197	0.975 [0.015–2.490]	0.209
Frequency of ART uptake	Frequent	213 (70.3)	90 (29.7)	1		1	
	Skip	44 (72.1)	17 (27.9)	1.094 [0.593–2.016]	0.774	0.975 [0.512–1.854]	0.938
Drinking of alcohol	No	79 (71.2)	32 (28.8)	1		1	
	Yes	178 (70.4)	75 (29.6)	0.961 [0.588–1.571]	0.875	0.718 [0.534–1.540]	0.718
Smoking/snuffing	No	251 (70.7)	104 (29.3)	1		1	
	Yes	06 (66.7)	03 (33.3)	0.829 [0.203–3.376]	0.793	1.076 [0.231–5.007]	0.926
Bath before and immediately after sexual intercourse	No	129 (76.3)	40 (23.7)	1		1	
	Yes	128 (65.6)	67 (34.4)	0.592 [0.373–0.940]	0.026	0.458 [0.272–0.772]	*0.003

### Clinical factors associated with the transmission of STI among PLWHIV infection

Out of the six clinical parameters examine for their association with STI transmission, non- showed any significant association with sexually transmitted infection as shown in Table 11.

**Table 11:** Clinical factors associated with the transmission of sexually transmissible infection among PLWHIV.

	Category	Negative (%)	Positive (%)	COR [95% CL]	*p*-value	AOR [95% CL]	*p*-value
High blood	No	245 (70.4)	103 (29.6)	1		1	
	Yes	12 (75.0)	04 (25.0)	1.261 [0.397–4.002]	0.694	1.255 [0.392–4.022]	0.702
Diabetes	No	255 (70.4)	107 (29.6)	1		1	
	Yes	02 (100)	0 (0.0)	677866521.4 [0.000]	0.999	709387934.4 [0.000]	0.999
ABO group	A	80 (72.7)	30 (27.3)	1		1	
	AB	05 (83.3)	01 (16.7)	0.781 [0.464–1.315]	0.353	0.772 [0.457–1.304]	0.334
	B	47 (74.6)	16 (25.4)	0.417 [0.048–3.646]	0.429	0.402 [0.046–3.527]	0.411
	O	125(67.6)	60 (32.4)	0.709 [0.372–1.352]	0.297	0.735 [0.383–1.410]	0.354
Rhesus type	Rh-	26 (76.5)	08 (23.5)	1		1	
	Rh+	231 (70.0)	99 (30.0)	0.718 [0.314–1.641]	0.432	0.712 [0.309–1.639]	0.424
HBV vaccine	No	253 (70.5)	106 (29.5)	1		1	
	Yes	04 (80.0)	01 (20.0)	1.676 [0.185–15.170]	0.646	1.760 [0.192–16.123]	0.617
Blood transfusion past 3 months	No	252 (71.0)	103 (29.0)	1		1	
	Yes	05 (55.6)	04 (44.4)	0.511 [0.135–1.941]	0.324	0.535 [0.139–2.057]	0.363

## Discussion

Sexually transmitted infections (STIs) are a major public health issue, particularly in Africa (20). These infections play a key role in HIV transmission by penetrating protective mucosal barriers and attracting vulnerable immune cells. Co-infection with HIV may even lead to serious health complications including the possibility of ART-drug resistance (21). However, their inclusion as part of the routine healthcare screening package just like viral load assessment is yet to be implemented in Cameroon. As such most asymptomatic STI infected individuals end up being undetected during their routine clinical management. Therefore, to properly address and manage STIs and the health hazards they bring, it is imperative to understand their prevalence in PLWHIV. This study therefore was aimed at determining the seroprevalence of syphilis, chlamydia, and HBV among PLWHIV who came from both urban and rural areas attending the different hospitals where enrolment was being done. Additionally, it aimed to assess the ‘risk factors for associated with STI transmission within the study population. Nevertheless, due to political instability and civil unrest in the area, a hospital-based cross-sectional design was adopted instead of a community-based survey. This limits the inclusion of PLWHIV who do not access care through formal health facilities, introducing potential selection bias This possibly may have an influence on the study outcome.

The overall observation of our study shows that STI seroprevalence is high among PLWHIV infections with syphilis taking the first place with distinction followed by HBV and finally chlamydia infection. PLWHIV infection came from rural areas recorded the highest seroprevalence of these STIs. The findings of our study are in line with similar studies conducted in Bangladesh and a narrative review conducted by Jenkins et al. (14, 22). This may be because access to proper healthcare in most of the rural areas is a problem as most of the health facilities have been shut down or better still abandoned due to the anglophone crisis. The few facilities that are operational lack the necessary resources to deliver proper medical care to patients. Despite the development and piloting of numerous STI interventions, very few have been assessed for scalability or operationalization in rural settings. Our study also shows that few of the STI infected individuals had more than one infection, with individuals who came from the urban area dominating. This may be due to greater anonymity, greater mobility resulting in more sexual partners, and larger population density are all characteristics that raise the risk (23). However, this finding warrants the need for future studies assessing multiple infections among individuals from rural and urban communities.

Also, it was realized that despite the increase in the level of education in urban areas, STI seroprevalence was higher among those with secondary (79.6%) and tertiary (100%) education when compared with those participants who came from rural areas. This finding is contrary to the findings of Painters et al. (24) who reported that increase levels of education are generally associated with reduced STI seroprevalence in urban areas because education enhances knowledge about STIs, improves decision-making regarding sexual health, promotes the adoption of protective measures like condom use, and can foster better access to healthcare services. But it should be noted that the protective impact of education can differ depending on demographic factors like race, and for it to be completely effective, integrated interventions that address social determinants and contextual-level factors may be needed. However, the observations of our study may be due to greater opportunity for unsafe sexual activity, higher population density, and socioeconomic differences (25). The high STI seroprevalence observed among individuals with low level of education who came from rural areas is in line with the finding of similar study conducted in Vietnam (26).

The seroprevalence of STIs also showed an association with occupation and varied with the type of occupation as seen in our findings. Individuals who testified of being farmers coming from rural communities recorded the highest seroprevalence of STI infection. This finding is in line with the 2013 report of Ontario HIV treatment network (OHTN) (27). In their report, they suggested that it is possible that migrant farm workers have false beliefs about HIV infection and safer sex, as well as cultural values, beliefs, and practices that could make HIV acquisition easier. Additionally, a direct reaction to the everyday stressors of the migrant farming lifestyle, such as being cut-off from family and community, lacking social support, and having no control over living and working conditions, may also be more risky behaviour exposing them to risky sexual behaviours that increases their susceptibility to STI infections (27). Nevertheless, the high seroprevalence of 100% STI seen among individuals who were civil servants and students coming from the urban center may have been due to the small sample size used in our study. Therefore, future studies with a much larger sample size are needed to effectively evaluate the association between occupation and STI morbidity. The high seroprevalence of STI (89.5%) among participants using flushing toilets who came from urban area may be because of the fact that a disproportionate amount of the population in that group uses this system of toilet. This finding is contrary with that of a previous studies which reported that this system of toilet improves health as well as environmental protection (28). However, it was made clear that their effectiveness relies on strict sanitation and maintenance. Nevertheless, the increased seroprevalence of STI among pit toilet users who came from rural areas compared to those from urban areas may be attributed to the possibility of insufficient sanitation of these toilets among users. This result is in conformity with the reports of Twisk et al. and a couple of other studies (28–30). This finding is a call for concern and future research is required to examine how these two toilet systems may contribute to the spread of STIs.

The highest seroprevalence (80.3%) of STI observed among individuals from urban area living close to a health facility (<1 h drive) is contrary to the findings of other studies such as Haley et al. (29). Nevertheless, this finding is in line with the reports of Handebo in Ethiopia (30). This may not be directly caused by proximity, but rather by a number of factors, such as a higher chance of developing symptoms or engaging in high-risk behaviour (30, 31). Nevertheless, studies have reported mixed results regarding STI prevalence and proximity to health facilities.

Multivariate analysis in our study shows that the likelihood of female acquiring STI infection was lower when compared to men. This finding is contrary to the observations of other studies that reported women to be at higher risk of contracting STI than men since women are more prone to STI infections because they have a greater mucosal surface area exposed during sexual intercourse (32). The cervix at the distal end of the vagina connects to the uterus, endometrium, fallopian tubes, and ovaries, which are all part of the upper genital tract. STIs can cause a range of symptoms and effects at various locations along the female reproductive system, such as infertility, pelvic inflammatory disease (PID), vaginitis, and genital ulcer disease if not properly managed (33). The result of our finding may be as a result of one of the following multiple factors as reported by Anguru et al. (34): low levels of sexual activity overall, higher rates of consistent condom use by both male and female partners and the lack of high-risk behaviours like substance abuse or low number of individuals having multiple partners within the study population. Nevertheless, the likelihood of participant who takes a bath before and after having sex to acquire STI was also low. This observation is in contrary with the findings of other studies (35). Washing after sex may marginally increase the risk of STIs by possibly harming delicate membranes and removing protective fluids. Using condoms or other barrier methods correctly and consistently is the most effective way to prevent STIs. Risk can be decreased by maintaining proper hygiene, such as washing your hands and urinating after having sex, as well as by being open and honest with your partners about your sexual history and health (16, 35, 36). This may be a contributing factor to why individuals who testified of having sexual intercourse 1–3 times a week showed lower risk to STI infection. This finding is consistent with the finding of Siziba et al. (37) who reported that monogamous relationships with a small number of partners are less likely to result in STIs than frequent or multiple partners. Further reducing the likelihood of infection exposure could be achieved by this group’s consistent condom use or monogamous behaviour (37).

Even though the finding is in line with the findings of several studies (8, 10, 16, 33, 38, 39), it should be noted that STI transmission vary across the different regions in Cameroon (16, 36). Although there are intervention strategies, they are usually impeded by high rates of risky behaviour adoption, low awareness and knowledge levels, and a widespread stigma associated with STIs. Thus, it is crucial for global intervention, prevention, and control strategies to raise people’s awareness of STIs and how they affect their attitudes, knowledge, risky behaviours, and perceptions. Data on risky behaviours, perceptions, knowledge, awareness, and attitudes among developing countries are scarce, though, particularly among PLWHIV in Cameroon.

### Strengths and limitations of the study

This study happens to be the first of its kind evaluating the presence of all three infections among PLWHIV in this locality. It highlights the importance and need of incorporating STIs screening as part of the routine health package for PLWHIV seeking HIV treatment at HIV treatment centres instead of just the routine viral load evaluation. However, the study has a couple of limitations. Due to the political instability and civil unrest in the area, a hospital-based cross-sectional design was adopted instead of a community-based survey. This limited the inclusion of PLWHIV who do not access care through formal health facilities, introducing potential selection bias. Consequently, the facility-based sampling strategy may not fully represent the broader population of PLWHIV in Meme Division, especially those from remote or underserved areas. Therefore, the generalizability of the findings to all rural and urban PLWHIV should be interpreted with caution. Additionally, the small sample size and focus on a single division (Meme) may reduce the applicability of results to other regions in Cameroon or sub-Saharan Africa. Reliance on self-reported data for sensitive variables such as sexual behaviour and socio-economic status introduces the potential for reporting bias. Finally, the lack of longitudinal follow-up precludes any assessment of temporal trends or progression of STI co-infections. Moreover, due to the cross-sectional nature of this study, all associations between risk factors and STI prevalence should be interpreted as correlational rather than causal. Future longitudinal studies are required to determine the directionality of these relationships. The fact that only antibody test was used in our analysis with no confirmatory test could be a possible limitation to this study as this may lead to an increased number of false positive results due to the cross reaction of antibodies. Therefore, future studies utilizing a confirmatory test such as the polymerase chain reaction which detects the presence of these antigens is necessary to determine the true prevalence of these infections.

## Conclusion

This study depicts a high seroprevalence of STI among PLWHIV in Meme division with syphilis dominating, followed by HBV and finally chlamydia. Individuals who came from rural communities recorded the highest seroprevalence of these infections with an exception of HBV which recorded an increase seroprevalence among individuals who came from urban areas with sex, having multiple sexual partners and sex frequency being the potential factors associated with STI morbidity. Therefore, routine screening of STIs needs to be adopted as part of the health packages for PLWHIV and, larger community-based studies looking at the prevalence of these infections among PLWHIV are needed as well as studies evaluating the effect of the interaction between these infections on ART-drug resistance.

## Data Availability

The raw data supporting the conclusions of this article will be made available by the authors, without undue reservation.
